# Importance of feasibility assessments before implementing non‐interventional pharmacoepidemiologic studies of vaccines: lessons learned and recommendations for future studies

**DOI:** 10.1002/pds.4081

**Published:** 2016-09-07

**Authors:** Corinne Willame, Laurence Baril, Judith van den Bosch, Germano L. C. Ferreira, Rachael Williams, Dominique Rosillon, Catherine Cohet

**Affiliations:** ^1^ Business and Decision Life Sciences on behalf of GSK VaccinesWavreBelgium; ^2^ GSK VaccinesWavreBelgium; ^3^ Pallas Health Research and ConsultancyRotterdamthe Netherlands; ^4^ P95 Pharmacovigilance and Epidemiology ServicesLeuvenBelgium; ^5^ Clinical Practice Research DatalinkLondonUnited Kingdom

**Keywords:** pharmacoepidemiology, feasibility assessment, lessons learned, study design, recommendations, toolbox

## Abstract

**Purpose:**

Investigational and marketed vaccines are increasingly evaluated, and manufacturers are required to put in place mechanisms to monitor long‐term benefit–risk profiles. However, generating such evidence in real‐world settings remains challenging, especially when rare adverse events are assessed. Planning of an appropriate study design is key to conducting a valid study. The aim of this paper is to illustrate how feasibility assessments support the generation of robust pharmacoepidemiological data.

**Methods:**

Following an initiative launched by the International Society for Pharmacoepidemiology in May 2014, a working group including members of the private and public sectors, was formed to assess the value of conducting feasibility assessments as a necessary step before embarking on larger‐scale post‐licensure studies. Based on five real‐life examples of feasibility assessments, lessons learned and recommendations were issued by the working group to support scientific reasoning and decision making when designing pharmacoepidemiologic vaccine studies.

**Results:**

The working group developed a toolbox to provide a pragmatic approach to conducting feasibility assessments. The toolbox contains two main components: the scientific feasibility and the operational feasibility. Both components comprise a series of specific questions aimed at overcoming methodological and operational challenges.

**Conclusions:**

A feasibility assessment should be formalized as a necessary step prior to the actual start of any pharmacoepidemiologic study. It should remain a technical evaluation and not a hypothesis testing. The feasibility assessment report may facilitate communication with regulatory agencies toward improving the quality of study protocols and supporting the endorsement of study objectives and methods addressing regulatory commitments. © 2016 The Authors. *Pharmacoepidemiology and Drug Safety* published by John Wiley & Sons Ltd.

## Introduction

In pre‐licensure studies, rare adverse effects of drugs and vaccines may go undetected. This safety concern drives regulatory authorities and public health agencies to put in place mechanisms to monitor the longer term and real‐life safety and benefit of products as well as their added value for public health. Worldwide, the regulatory environment is ever‐evolving, increasingly complex and stringent, requiring a high level of compliance and scientific expertise from pharmaceutical companies. Several guidance and directives related to requirements for post‐marketing studies have been issued in Europe^1^, ^2^, ^3^ and in the United States (US)^4^, ^5^. Recently, some countries in other regions have also developed well‐defined local pharmacovigilance regulations (e.g. India^6^ or Brazil^7^).

Organizations like the Clinical Practice Research Datalink General Practice OnLine Database^8^ (CPRD GOLD) group have seen an increase in the number of database access requests to support the development of post‐approval drug safety studies (68 protocols submitted to the Independent Scientific Advisory Committee in 2014, compared to 30 in 2011).

Since vaccines are generally administered to healthy populations, benefit–risk monitoring at the individual and community level are crucial. Entire birth cohorts of infants or children are targeted by a vast range of vaccines. For instance, approximately 85% of the vaccines distributed by GSK are intended for the pediatric population (2014 unpublished GSK internal data). Also, in comparison to drugs used to treat existing diseases, vaccines are administered on a much larger scale in the population. Currently, around 72 million individuals worldwide have received the Human Papillomavirus (HPV) vaccine^9^, and around 40% of people in the US are immunized with seasonal influenza vaccines each year^10^. These numbers raise the potential for very rare adverse events to be detected by surveillance.

Another specific feature of vaccines is that the immune response triggered by immunization can be expected to generate non‐serious adverse reactions, such as fever or pain at injection site^11^, in a not insignificant proportion of recipients and the level of acceptance of these and other side effects in, for example, healthy children is very low. Finally, the introduction of new technologies may raise some concerns: for example, novel adjuvanted vaccines have been raising questions related to their safety profile and their theoretical capacity to cause autoimmune adverse reactions^12^.

When vaccines are marketed, tolerance for any risk of serious adverse events (SAE) is extremely low. In addition, reliable risk estimates for very rare (incidence range: <1 to 10/100,000 person‐years) safety outcomes cannot usually be provided by pre‐licensure clinical studies. Pharmacoepidemiologic (PE) studies are often seen as the best option to deliver evidence of safety post‐licensure^13^. It is challenging to design sufficiently robust PE studies to generate reliable evidence on rare safety outcomes in real‐life settings. Depending on the specific research question, and for an acceptable level of evidence quality, careful attention must be given to the optimal study design and data source (e.g. field studies involving primary data collection vs. studies using large healthcare databases). In addition, because of constraints such as low vaccine uptake in certain regions/sub‐populations, special populations with underlying conditions (e.g. pregnancy, comorbidities), governance and/or resource issues, prospective field studies cannot be implemented or deliver results rapidly. Retrospective studies using electronic medical records become a more time and cost‐efficient alternative. On the other hand, because of their retrospective nature, such studies may have limitations related to exposure and/or outcome ascertainment. Regardless of the data source, the study design should consider the adequacy of the sample size (i.e. power), minimization/control of bias and confounding, accuracy of exposure information, and degree of specificity of the outcome assessment^13^. These aspects are challenged by ever‐increasing expectations with respect to the quality of research voiced by the scientific community, the regulatory agencies, vaccine recommending bodies, and the public at large.

Observational research in epidemiology/pharmacoepidemiology is supported by several guidelines such as the Guidelines for Good Pharmacoepidemiological Practices (GPP)^14^, the STROBE and RECORD recommendations^15^, ^16^, PRISMA statements^17^, guidelines for good database selection^18^, and European Network of Centres for Pharmacoepidemiology and Pharmacovigilance^3^ (ENCePP) guidelines. In addition to these, the likelihood of success of a study can be optimized by essential pre‐requisites such as a feasibility assessment or a pilot study^19^.

Based on vaccine examples of feasibility assessments, the objectives of this paper are (i) to demonstrate the value of conducting a formal feasibility assessment as a necessary step when planning and designing a safety and/or effectiveness study and (ii) to propose a toolbox and recommendations to support the scientific approach when assessing study feasibility.

## Methods

### Working group

In May 2014, the International Society for Pharmacoepidemiology (ISPE) launched a call for manuscripts^20^. The requirement was to establish a working group with members from different horizons to develop a manuscript addressing the role and value of non‐interventional pharmacoepidemiologic studies. This manuscript was prepared by seven volunteers from the private and public sectors and peer‐reviewed by members of the ISPE Special Interest Group in Vaccines (VAXSIG).

### Vaccine research studies—examples

Five post‐licensure studies were used as the basis for collecting key elements on their respective feasibility assessments (Table 1). The studies were all post‐licensure commitments fulfilling requirements from the Food and Drugs Administration (FDA) or European Medicine Agency (EMA). The need for a feasibility assessment was identified at an early stage of conceptualizing for each study. All studies were registered on www.clinicaltrials.gov and/or the ENCePP EU PAS (European post‐authorization studies) register^3^. These studies were selected by the seven working group members who all had substantially contributed to at least one of the studies (Table 1).

**Table 1:** Description of selected post‐licensure studies for which feasibility had been assessed

Study #	Vaccine	Study objective	Study design/setting	Registered at www.clinicaltrials.gov	ENCePP E‐Register number	Related publication
**1**	Cervarix^TM^ HPV‐16/18 vaccine	To assess the risk of spontaneous abortion after inadvertent exposure to HPV‐16/18‐vaccine during pregnancy	Observational cohort study in the CPRD database	NCT01905462	ENCePP id 3310	Baril *et al.*, 2015^22^
**2**	Cervarix^TM^, HPV‐16/18 vaccine	To assess the risk of autoimmune diseases in women aged 9–25 years within 1 year after the first vaccine dose	Observational cohort study in the CPRD database	NCT01953822	ENCePP id 4584	Submitted
**3**	Pandemrix^TM^, H1N1 pandemic influenza vaccine	To assess the risk of solid organ transplant (SOT) rejection	Retrospective self‐controlled case series in the CPRD database and HES	NCT01715792	ENCePP id7070	Cohet *et al.*, 2016^23^
**4**	Rotarix^TM^, rotavirus vaccine	To assess the association between Rotarix™ and intussusception in infants in the context of the mass vaccination initiated in 2006 in Mexico	Prospective active surveillance study in hospital setting	NCT00595205	NA	Vélazquez *et al.*, 2012^24^
**5**	Mosquirix™, Malaria vaccine	To determine baseline rates of pre‐defined diseases and meningitis leading to hospitalization or death	Prospective cohort field study in health care facilities	NCT02374450	NA	NA

CPRD: Clinical Practice Research Datalink database, ENCePP: European Network for Centers for Pharmacoepidemiology and Pharmacovigilance; HES: Hospital Episodes Statistics; id: identifier; NA: not applicable; NCT: National Clinical Trial. Note: All studies were approved by the respective ethics committees/ethical review boards.

### Feasibility assessment output

Working group members provided details of the feasibility assessment for each example, including both information known *a priori* (i.e. before the start of the study) and new evidence specifically generated by the feasibility assessment. These data were grouped according to three main topics: population, exposure, and outcome. The output of this exercise is summarized in Table 2.

**Table 2:** Summary of feasibility assessment outputs

Study (exposure, outcome)	Design criteria	Feasibility assessment outputs	
		What was known before the feasibility assessment?	What was found by conducting the feasibility assessment?
**Study** #**1** (HPV vaccine, spontaneous abortion)	Population and setting information	‐Pivotal clinical trial data showed a potential risk. ‐Target population for the vaccine has a specific age indication. ‐Previous field study negative or inconclusive. ‐Lack of comprehensive information in using the selected database (CPRD).	‐Deep understanding of the database (CPRD), for example benefit of using linked data sources. ‐Identified need for partnership with specialized company and expert panel in teratology.
	Exposure	‐Known vaccine coverage in the UK. ‐Immunization programme through schools in the UK.	‐Implementation of blinded procedure for exposure status during the case ascertainment.
	Outcome	‐Data on background rates of spontaneous abortion published in the literature. ‐Studies related to pregnancy outcomes and using CPRD and free text were published.	‐Development of algorithms with high PPV for case finding. ‐Need for a review of medical records and case ascertainment process with medical experts. ‐The database showed consistency in generating baseline data when comparing to literature.
**Study** #**2** (HPV vaccine, autoimmune diseases)	Population and setting information	‐Theoretical risk of autoimmune diseases with novel adjuvanted vaccine. ‐Target population for the vaccine has a specific age indication. ‐Similar studies already published, for example study conducted by other vaccine manufacturer, availability of algorithms for case finding in database study.	‐Agreement with regulatory authorities reached on a pre‐defined list of adverse events of special interest. ‐Identified need for partnership with specialized company and experts in the medical area of interest.
	Exposure	‐Known vaccine coverage in the UK. ‐Immunization programme through schools in the UK.	‐Implementation of procedure blinded to exposure status for case ascertainment.
	Outcome	‐Medical management of the outcome mainly in hospital/specialist settings.	‐Systematic literature review conducted to reinforce background incidence data. ‐The database showed consistency in generating baseline data when comparing to literature. ‐Development of specific algorithms for case finding using HES. ‐Need for a review of medical records and case ascertainment process with an expert panel.
**Study** #**3** (H1N1 pandemic influenza vaccine, solid organ transplant rejection)	Population and setting information	‐A signal emerged from real‐world use of the vaccine. ‐Target population for the vaccine is a high risk group. ‐Previous feasibility assessment on field study inconclusive.	‐Important proportion of missing data in the CPRD triggered need for collecting complementary information from GPs through questionnaire. However, lack of comprehensive information returned led to the use of HES as primary data source for case identification. ‐Use of CPRD to extract covariates (risk factors) information.
	Exposure	‐H1N1 mass immunization through GPs in the UK. ‐Known brand‐specific H1N1 vaccine coverage in the UK.	NA
	Outcome	‐Clinical complexity of the outcome involving numerous risk factors. ‐Medical management of the outcome mainly in hospital/specialist settings, questioning the appropriateness of using CPRD.	‐Development of specific algorithms for case finding, using HES as primary data source. ‐Time since transplantation identified as risk factor for solid organ transplant rejection, thus included as covariate in analyses
**Study** #**4** (Rotavirus vaccine, intussusception)	Population and setting information	‐A signal emerged from the real‐world use of a similar vaccine. ‐Target population for the vaccine has a specific age indication. ‐Availability of passive surveillance system for adverse events of special interest in Mexico.	‐Implementation of an active surveillance system.
	Exposure	‐Known vaccine coverage in Mexico.	NA
	Outcome	‐Medical management of the outcome in hospital settings.	‐Evaluation of the active surveillance system performed by an external company as part of a pilot study.
**Study** #**5** (Malaria vaccine, autoimmune disease, KD, meningitis)	Population and setting information	‐Theoretical risk of autoimmune diseases with novel adjuvanted vaccine. ‐Pivotal clinical trial data showed a potential risk of meningitis. ‐Literature reviews show scarcity of background rates for adverse events in SSA. ‐No existing databases in SSA thus need for prospective data collection.	‐Comprehensive literature review conducted to reinforce background incidence data. ‐Positive scientific opinion by experts or health agency on the proposed study protocol. ‐Identified need for partnership with specialized agency (HDSS). ‐Identified need for capacity building, for example know‐how in pharmacovigilance systems, medical diagnosis, laboratory capacities.
	Exposure	NA	NA
	Outcome	‐Multiple outcomes (AEs) of interest	‐Support of an expert panel for case ascertainment.

AE: Adverse Event; CPRD: Clinical Practice Research Datalink; GP: General Practice; HDSS: Health and Demographic Surveillance Sites; HES: Hospital Episode Statistics; HPV: human papillomavirus; KD: Kawasaki Disease; NA: Not Applicable; PPV: Positive Predictive Value; SSA: Sub‐Saharan Africa; UK: United Kingdom.

### Toolbox design

A pragmatic approach is proposed to support scientific reasoning and decision‐making in the initiation and development of the feasibility assessment. The toolbox consists of two main components addressing both scientific and operational feasibility, comprising a series of specific questions to help identify strengths/limitations and to fill data gaps on key elements of the anticipated study design.

**Figure 1:**
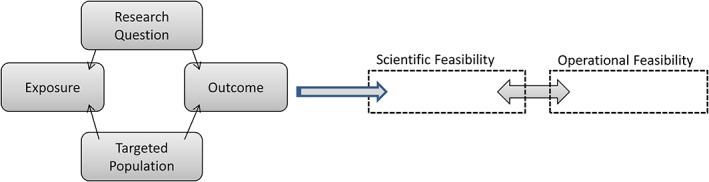
Schematic representation of the toolbox

The scientific feasibility component addresses aspects related to exposure, outcome, and target population. The operational feasibility focuses on medical governance, logistical constraints for the vaccine manufacturer, and the need for potential partnerships or collaborations. Figure 1 presents a schematic view of the proposed toolbox.

## Results

### Lessons learned

Each feasibility assessment includes specific lessons learned, actions, or implementations (Table 2).

#### **Study** # **1** (Exposure: HPV vaccine, Outcome: spontaneous abortion)

A field study with primary data collection was initiated in the US to assess this association. However, due to very low vaccine uptake, the target sample size (*n* = 450 subjects) could not be reached within the 2‐year time period requested by FDA to address the commitment. Given the time and resources that would have been necessary to prospectively accrue a sufficiently powered study population, an alternative retrospective database study in the CPRD GOLD database was proposed, as vaccine coverage in the United Kingdom (UK) was adequate to implement a post‐authorization safety study (PASS). However, the feasibility assessment showed a lack of sensitivity (high rate of false negatives) in the vaccination records. This issue was resolved by using a vaccinated control cohort with pregnancy onset after a sufficient post‐vaccination period to exclude any possible vaccine effect. In‐depth knowledge of the complexity of using the database was gained through the feasibility assessment, as well as through the actual study, which ultimately also benefited further studies with other exposure and/or outcome. Limitations related to this data source (e.g. lack of specificity of the exposure and outcome) were overcome by a modification of the cohort design, along with a detailed review of individual subject profiles and a case ascertainment by teratology experts blinded to vaccination status.

#### **Study** # **2** (Exposure: HPV vaccine, Outcome: autoimmune diseases)

Given the low incidence of the outcome in the target population of the vaccine, this study used a database design upfront. Pre‐defined list of autoimmune conditions and sample size requirements were agreed with regulatory authorities (FDA). A robust feasibility assessment was performed to define algorithms and assess their accuracy. The positive predictive value (PPV) of the algorithms was 69%, highlighting the need for a robust case ascertainment plan to increase clinical endpoint specificity. A combined approach using the data retrieved by the algorithms and a review of the medical electronic records in addition to the associated free text (e.g. hospitals discharge and notes from general practices) were performed to ensure adequate case validation.

#### **Study** # **3** (Exposure: H1N1 pandemic influenza vaccine, Outcome: solid organ transplant rejection)

The study was implemented following a stepwise feasibility approach. The first step investigated ways of implementing a field study. Extensive surveys were conducted in specific settings (national transplant registries and hospitals specialized in transplantation) within five countries (UK, France, Brazil, Canada, and Germany). However, low survey response rates and paucity of medical/vaccination data were identified. An extended follow‐up feasibility assessment was conducted in two of the five countries (UK and Brazil) using detailed site surveys to assess hospital type, standard of care, comprehensive patient information (compliance to treatment, drug regimen, history of infection…), and medical record linkage. Despite several limitations, the feasibility assessment concluded that a field study could be conducted in one country (feasible in Brazil, but not in the UK, mostly due to small sample size). However, concerns about methodology and generalizability of the results discouraged the launch of the study and suggested that a retrospective database study was preferable. A further feasibility assessment in the CPRD confirmed the need to develop robust algorithms as well as include additional linked data from the Hospital Episodes Statistics (HES) database and complementary information from general practices through standardized questionnaires.

#### **Study** # **4** (Exposure: Rotavirus vaccine, Outcome: intussusception)

A hospital‐based active surveillance system was implemented in Mexico to collect specific adverse events (AE). However, the active surveillance system showed inconsistencies in the enrollment of subjects over time. A feasibility assessment was initiated to ensure the performance of the active surveillance systems in the collection of two AEs of interest (intussusception and lower respiratory tract infections). In addition, the robustness of the data collection system was evaluated by the scientific validity of the results generated. The feasibility assessment was conducted as part of a pilot study in partnership with a company specializing in health information systems.

#### **Study** # **5** (Exposure: Malaria vaccine, Outcome: autoimmune diseases, Kawasaki disease, intussusception, meningitis, and other pre‐defined diseases)

The feasibility assessment performed in Sub‐Saharan Africa confirmed that a field study could be implemented through an existing network of health and demographic surveillance systems (HDSS) in African regions with low to moderate malaria endemicity. Missing key elements such as laboratory capacity, know‐how in pharmacovigilance and a need for an expert panel for case ascertainment for some of the endpoints were identified.

For each of these post‐licensure studies, the choice of the target country or geographical area was mainly dependent on the coverage of the vaccine of interest which further restricted a potential geographical scope. Moreover, due to the complexity of some outcomes or the lack of background/incidence data, a systematic review of the literature also had to be performed as a preliminary step.

### Recommendations: the toolbox

**Figure 2:**
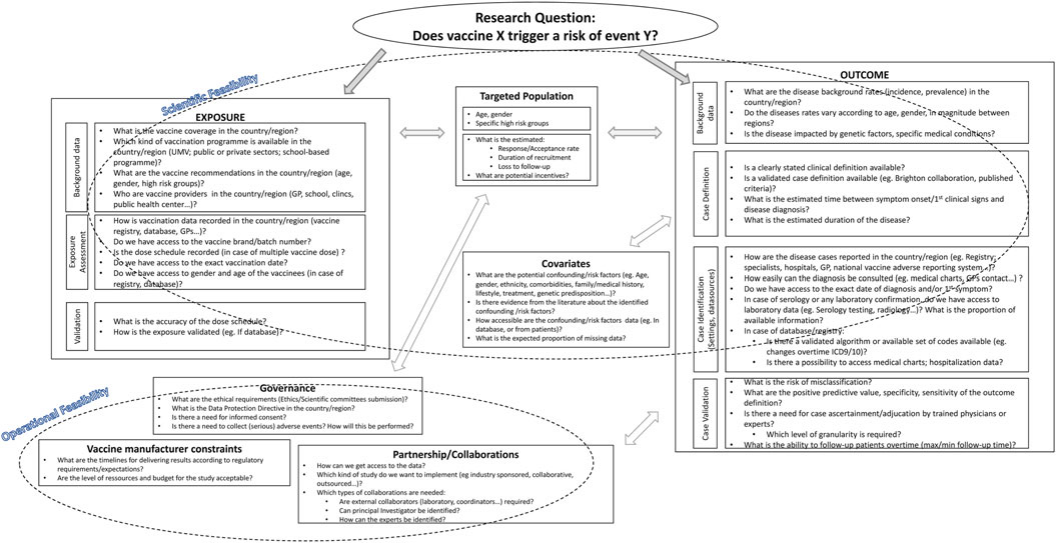
Toolbox

Based on the experience with these post‐licensure studies, the working group proposed recommendations in the form of a toolbox as a pragmatic approach (Figure 2) for the development of feasibility assessments to implement appropriate study designs. The assessment tool is divided into two mutually interdependent categories: (i) the scientific feasibility; and (ii) the operational feasibility. Within each category, multiple boxes define specific topics and include a series of questions. Answers for each of the questions are aimed at improving the knowledge around potential methodological challenges and provide information on the likelihood of success of the design approach (Box 1). The scientific feasibility focuses on the outcome and the exposure while the operational feasibility helps identifying logistical issues and needs (e.g. collaborations/partnerships, timelines, governance, ethical aspects). Before making a final decision on the future study design, it is recommended to perform this exercise for two or three different study designs, including a field and a database study, if relevant.

**Box 1**

Addressing scientific feasibility questions helps to define the appropriate study design and methodology:

- What is the most appropriate study design: prospective or retrospective; type of specific design, for example cohort (historical, concurrent, unmatched, matched, propensity scores), case–control (unmatched, matched, test‐negative), and case‐only (self‐controlled case series, case coverage)?
- What is the most appropriate data collection strategy: primary (field study) or secondary data collection (large healthcare database)?
- What is an adequate risk period?
- Is a comparator required; if so, what is an adequate control group?
- What is the required sample size?
- What are the most appropriate statistical methods:
  - To control for bias and known confounding factors;
  - To take into account potential unknown or unmeasured confounding factors;
  - To control for missing data?
  - To perform sensitivity analyses
- What are the inclusion/exclusion criteria?
- What are the expected limitations of the study?

Box 1 developed based on the European Network of Centres for Pharmacoepidemiology and Pharmacovigilance (EnCePP) guidelines^21^.

## Discussion

It is becoming routine that vaccine manufacturers are requested by regulatory authorities to perform specific studies to assess vaccine safety or effectiveness/impact. However, in some cases, the suggested designs may be unrealistic from an implementation perspective.

The rationale, choice of study design, and implementation of an informative and meaningful PE study require consideration of several important factors. The advantages and limitations of using secondary (existing electronic healthcare data) or *de novo* field/primary data collection should be clearly stated and documented. In addition, a critical feasibility assessment should be considered and undertaken as a first step before embarking on a larger study.

For each of the study objectives, the degree of granularity of the feasibility assessment needs to be tailored depending on the available information. Based on the feasibility outcomes of the five examples of post‐licensure studies for which a feasibility assessment was performed, we developed a toolbox to guide researchers in the design and implementation of a future study. Initially, we focused on technical and methodological aspects and on the understanding of the limitations of the available data (e.g. such as data accuracy and completeness, missing information), impact of existing known confounding factors, and opportunity for linkage with complementary data sources. Subsequently, we considered the feasibility assessment as a ‘pilot study’ to gain more insight into the specificity and sensitivity of the definition of the outcome of interest, data management flows, and external potential constraints (e.g. such as need for expert consultations, regulatory timelines, governance aspects).

Nevertheless, and importantly, the focus of the feasibility assessment should remain a technical evaluation and not a hypothesis testing. In our examples, the feasibility assessments were a ‘dig‐deeper evaluation’ to understand the data content in the first place and secondly, to plan the future study to answer the research question successfully. For instance, in study #2, a full ascertainment of autoimmune disease cases was performed by a physician on a sub‐sample of eligible cases to ensure a high PPV which was critical for the study's internal and external validity. In study #3, although a reasonable likelihood of success for the proposed field study was predicted, the representativeness and generalizability of the results were questionable. In addition, the feasibility assessment highlighted some limitations, such as lack of accurate reporting of the outcome of interest, which required development of an alternative study design. To date, the clinical definition criteria or diagnostic codes used to identify outcomes and exposures are not always included in scientific publications. However, the recent RECORD statements^16^ recommend a systematic reporting of codes and algorithms to classify exposure, outcome, and confounders which will facilitate study outcome comparisons. Finally, the studies succeeded because of a strong collaboration/partnership with external experts as well as database owners. The roles and responsibilities of each of the stakeholders were clearly established at the time of the feasibility assessment.

Feasibility assessments are critical to ensure that the research question is adequately addressed and timely generates the expected robust evidence to support decision making. In the case of an inconclusive assessment, rational and appropriate answers are provided such as a proposal for a mitigation plan or an acknowledgment of missing information in a risk management plan. These answers are generally endorsed by regulatory agencies.

Feasibility assessments can constitute a constructive first step in discussions with regulators to define how to obtain the expected best possible and timely evidence. Decisions on statistical power and sample size, endpoints and clinical case definitions, or means of adjustment for bias and confounding, as well as adequacy of the proposed study design to meet the study objectives, can be agreed early on, thus potentially avoiding multiple study protocol review rounds and potential future amendments. Moreover, this would allow adapting timelines from the time of the study protocol development to the reporting and interpretation of the study results to be realistically planned and communicated. This process should ultimately improve the quality of study protocols, accelerating the endorsement process by regulatory authorities and ethical committees, and in turn, the start of the actual study. Ideally, the feasibility assessment report should remain publicly accessible for consultation and considered as an *ad‐hoc* component of the study report (e.g. as a supplementary material with the study protocol and report registration and/or publication).

The above recommendations are based on examples of post‐licensure safety studies. However, effectiveness or burden of disease studies would benefit from the same proposed toolbox, which can be used as a roadmap to guide scientific reasoning when designing an observational study.

## Conclusion

With this report, the working group wishes to highlight and share recent experiences with feasibility assessments performed in the context of addressing commitments from regulatory authorities. A toolbox was designed to support the scientific reasoning when developing an observational study. In our examples, feasibility assessments led to a successful completion of the actual studies. Benefits of collaboration between industry research teams, clinical experts, and database owners were largely acknowledged. Our final recommendation would be to formalize the feasibility assessment as a first step of a larger‐scale study and as a complementary approach to existing guidelines (e.g. GPP, RECORD, good database selection^17^, ENCePP etc.). The ultimate goal of this pragmatic approach is to contribute to advancing knowledge in pharmacoepidemiology and increasing public confidence in how the safety profile of licensed vaccines is evaluated.

## Conflict of Interest

All authors have completed the ICMJE uniform disclosure form at www.icmje.org/coi_disclosure.pdf and declare: LB, DR, and CC are employed by, and own stock options in, the GSK group of companies. CW is a consultant from Business and Decision Life Sciences to the GSK group of companies. RW reports that CPRD is a research organization that conducts commissioned research for pharmaceutical companies and other organizations, including the GSK group of companies. JvB reports grants from the GSK group of companies, Crucell, Sanofi Pasteur, and European Centre for Disease Prevention and Control, outside the submitted work. GF report grants from the GSK group of companies, outside the submitted work.

Cervarix, Mosquirix, Rotarix, and Pandemrix are trademarks of the GSK group of companies.

**Key Points**

- Generating evidence in real‐world settings remains challenging, especially when assessing rare adverse events. Large sample sizes and accurate data sources are often required to assess the association between vaccine exposure and rare adverse outcomes.
- Feasibility assessments are necessary in planning and designing robust pharmacoepidemiologic studies, to highlight study strengths and limitations and facilitate informed decision‐making on study design.
- Based on real‐life examples, a toolbox was designed to support the scientific approach when performing feasibility assessments.
- Feasibility assessment reports could be used when addressing regulatory requests for post‐licensure studies, allowing an evidence‐based discussion and reinforcing a continuous collaboration between interested parties.

## Funding

This review was sponsored and funded by the International Society for Pharmacoepidemiology (ISPE). Co‐authors were involved in all stages of the review and manuscript development.
